# The defect feature extraction of ultrasonic phased array detection based on adaptive region growth

**DOI:** 10.1371/journal.pone.0297206

**Published:** 2024-01-25

**Authors:** Zhe Wang, Shuai Li, Chao Zhang, Xiahui Li, Haonan Long, Xianming Zhu

**Affiliations:** 1 School of Automotive Application, Hunan Automotive Engineering Vocational College, Zhuzhou, China; 2 Children’s Health Department, Changsha Maternal and Child Health Hospital, Changsha, China; 3 College of Electrical and Information Engineering, Hunan University, Changsha, China; Chitkara University Institute of Engineering and Technology, INDIA

## Abstract

An ultrasonic phased array defect extraction method based on adaptive region growth is proposed, aiming at problems such as difficulty in defect identification and extraction caused by noise interference and complex structure of the detected object during ultrasonic phased array detection. First, bilateral filtering and grayscale processing techniques are employed for the purpose of noise reduction and initial data processing. Following this, the maximum sound pressure within the designated focusing region serves as the seed point. An adaptive region iteration method is subsequently employed to execute automatic threshold capture and region growth. In addition, mathematical morphology is applied to extract the processed defect features. In the final stage, two sets of B-scan images depicting hole defects of varying sizes are utilized for experimental validation of the proposed algorithm’s effectiveness and applicability. The defect features extracted through this algorithm are then compared and analyzed alongside the histogram threshold method, Otsu method, K-means clustering algorithm, and a modified iterative method. The results reveal that the margin of error between the measured results and the actual defect sizes is less than 13%, representing a significant enhancement in the precision of defect feature extraction. Consequently, this method establishes a dependable foundation of data for subsequent tasks, such as defect localization and quantitative and qualitative analysis.

## Introduction

Ultrasonic phased array technology, a prominent tool in industrial non-destructive testing, is extensively employed for detecting and imaging internal defects such as cracks, holes, and other anomalies in components. B-type detection is predominantly utilized in this context [1]. This technique surpasses traditional A-type detection by displaying multiple A-type waveform data on a single image. Moreover, A-wave data can be transformed into RGB images through data processing, aiding inspectors in deducing defect location and size [2–4]. Nevertheless, B-scan images from ultrasonic phased arrays often contain considerable noise due to factors like environmental noise, component geometry, and detection equipment. This noise compromises image quality and impedes defect identification, necessitating targeted image processing for precise defect extraction and quantification.

Three primary methods exist for defect feature extraction in ultrasound phased array B-scan images, each with its own set of accomplishments. The first is the threshold segmentation method. For instance, Kun Wu implemented an approach to automatically extract regions of interest (ROIs), using Otsu-AWDO for segmenting specific interfaces in IMC [5]. Nithya demonstrated kidney stone detection using an artificial neural network, with segmentation facilitated by a multikernel k-means clustering algorithm [6]. Ameer S achieved optimal histogram thresholding through a comparative element analysis for image segmentation [7]. This method is straightforward and computationally efficient, performing well with images where target and non-target regions are distinctly recognizable. However, its effectiveness diminishes when there is minimal contrast in pixel values, making appropriate threshold selection challenging. The second method involves convolutional neural networks (CNN). Siyuan Z developed a dataset of 1063 ultrasound images, employing deep CNN networks and residual networks for processing the IQA model, thereby deriving a medical image quality assessment method [8]. CNN are adept at automatically learning image features, handling large datasets, and learning rapidly. Nonetheless, they are computationally intensive, demand significant resources, and require high-quality original data. The third method, region segmentation, has been explored in various studies. Hu et al. combined mathematical morphology with iterative methods to extract defects in prefabricated holes and cracks [9], while Kozegar et al. utilized adaptive region growing and edge variable models for segmenting 3-D breast ultrasound images [10]. Although these methods optimize segmentation and feature extraction in traditional ultrasonic images, their performance is less effective in ultrasonic phased array B-scan images, where distinctions in gray levels are less apparent. The blending of initial waves, defect echoes, bottom loops, and noise leads to gray value overlap, complicating segmentation. Additionally, commonly used segmentation algorithms, such as the seed region growth algorithm, face challenges in adaptive seed point selection due to issues with seed point quantity and distribution, rendering them unsuitable for B-scan images.

Therefore, this study introduces an efficient, accurate, and flexible defect extraction method tailored for ultrasonic phased array B-scan images. This paper presents a feature extraction process based on adaptive region growth for ultrasonic phased array detection images. The proposed algorithm automatically and accurately extracts features like defect area, perimeter, perimeter-to-area ratio, and axis dimensions. The paper is structured as follows: initially, the basic principles of the algorithm are discussed. This is followed by a detailed implementation, where B-scan images are converted from RGB to gray scale, reducing computational load. Bilateral filtering is applied for noise removal. Automatic region growth is achieved through seed point acquisition, threshold updating, and region merging. Morphological processing enhances accuracy and facilitates defect extraction. The extracted feature values are then analyzed. Lastly, the efficacy and precision of the method are substantiated through comparative experiments. By integrating attributes of B-type ultrasonic phased array images, this algorithm advances traditional image segmentation and extraction techniques, significantly boosting the reliability and efficiency of ultrasonic phased array detection. Its application is crucial in equipment life assessment, safety evaluation, reliability analysis, fracture assessment, failure analysis, and non-destructive evaluation.

## Algorithm principle

**Fig 1:**
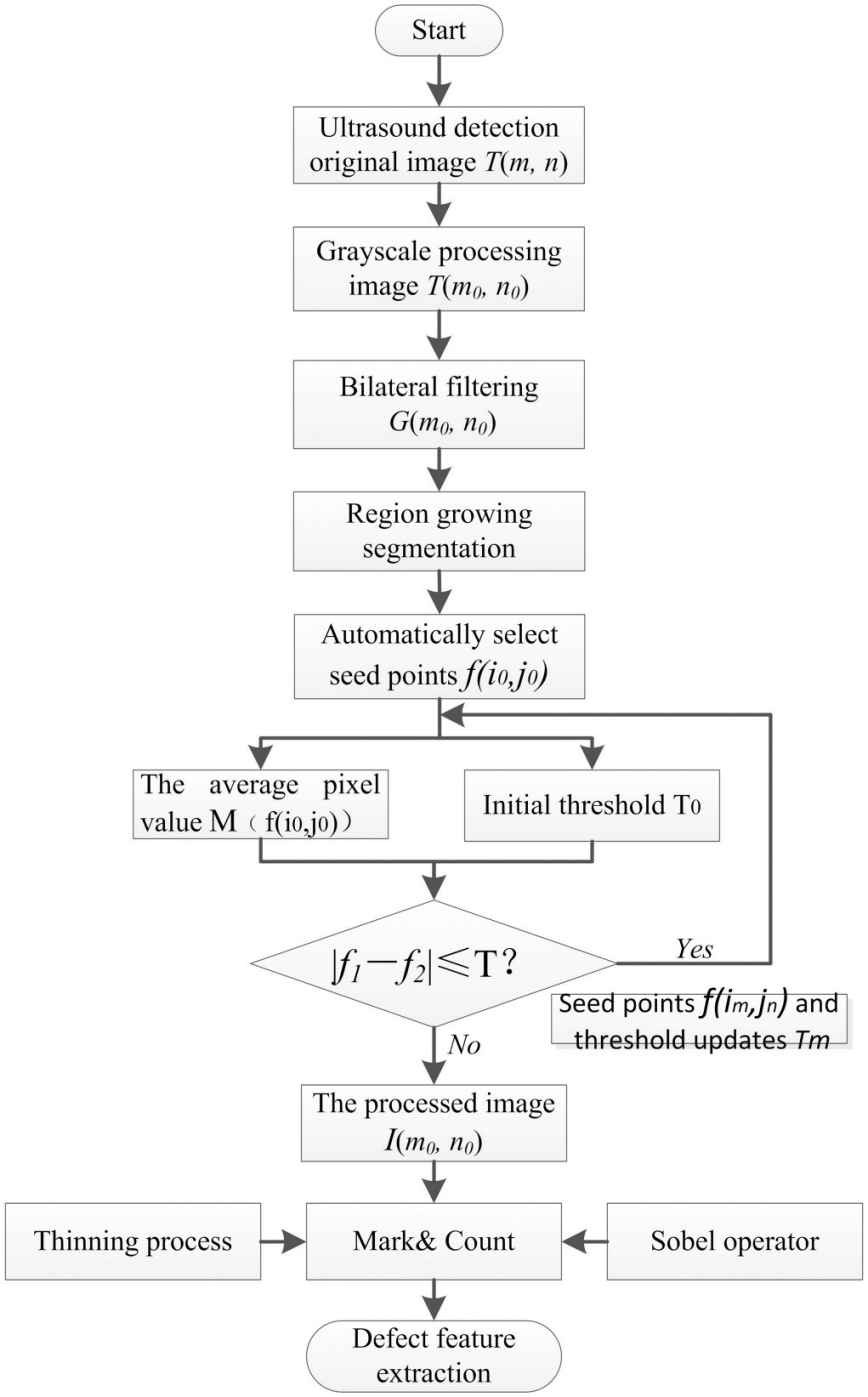
Defect feature extraction block diagram.

Obtaining geometric defect features from ultrasonic phased array B-scan images is the key to defect extraction and quantification [11]. The last acquired ultrasonic phased array B-scan image is marked by eight-connectivity discrimination methods for defect and contour tracking through image preprocessing, bilateral filtering, automatic region growth, mathematical morphological processing, etc. Geometric features such as perimeter, area, perimeter-area ratio, and long and short axes are selected to quantify and evaluate the accuracy of different methods for extracting defect features through numerical results and statistics. Fig 1 shows the specific process.

### The dataset

The ultrasonic phased array device is connected to MATLAB via USB, and the detecting device collects data from the B-type ultrasonic phased array test block and saves them as text documents, which are uploaded to the software in real time. The text document is converted into a numerical matrix of 128 × 400 in uint8 format by program calculation. The RGB value is assigned to the matrix value, the color is filled using the *pcolor* function, and the final detection image is formed by interpolation. The number of pixels in the image is 51200, and by identifying the number of pixels in the target area, the real value of each feature can be obtained according to scale conversion.

### Data processing

The images were only reformatted and normalized. Then, the path to the invoked image data was set, and the proposed algorithm was generally designed to work on the raw data; therefore, no further preprocessing was performed on the images. The raw data of the image and the processing code are already visible on GitHub (github.com/Sonyk700/code) and shown in the S1 File.

### Image preprocessing

The defect image obtained by the ultrasonic phased array detection system is a color image. However, the algorithm for color images is complex and inefficient during actual processing. Grayscale processing is needed to separate single-channel images to facilitate the extraction of defect characteristics. The grayscale image shows the depth of color. Its grayscale value is 0 to 255. 0 is black and 255 is white, and higher values are brighter. Grayscale is the projection on three channels of color image *R* (Red), *G* (Green), and *B* (Blue). As shown in Eq 1, the specific gray value can be obtained using the weighted average method [12].


Gray(i,j)=0.299×R(i,j)+0.578×G(i,j)+0.114×B(i,j) (1)

### Bilateral filtering

To better segment the target region of subsequent ultrasonic images and extract the target region of ultrasonic images for feature analysis, the ultrasonic images require preprocessing, such as filtering and denoising, before ultrasonic image segmentation.

Bilateral filtering removes noise and preserves edges, and it is a nonlinear filtering algorithm with a dual filtering function [13]. Bilateral filtering is a filtering function containing two Gaussian bases; thus, it not only considers the geometric proximity of each pixel value in the image but also considers the similarity of each pixel value in terms of brightness. The smooth image is obtained by adaptive filtering through the nonlinear combination of the two. The core idea of bilateral filtering is to multiply the Gaussian function related to the spatial distance (the Euclidean distance between the current point and the center point) with the Gaussian function related to the pixel distance (the absolute value of the difference between the pixel value of the current point and the center point). The principle expression of bilateral filtering is given in Formula 2.

$$\omega \left(i,j,k,l\right)=\text{exp}\left(-\frac{{\left(i-k\right)}^{2}+{\left(k-l\right)}^{2}}{2\sigma_{d}^{2}}-\frac{\left||I\left(i,j\right)-I{\left(k,l\right)}^{2}|\right|}{2\sigma_{r}^{2}}\right)$$ (2)

where *σ*_*d*_ is the standard deviation in the spatial domain, *σ*_*r*_ is the standard deviation of the value range, (*i*, *j*) is the position of the current pixel point, (*k*, *l*) is the position of the central pixel point, *I*(*i*, *j*) is the pixel value of the current point, and *I*(*k*, *l*) is the pixel value of the central point. Thus, the mathematical form of the spatial domain Gaussian function is given as follows:

$$exp\left(-\frac{{\left(i-k\right)}^{2}+{\left(k-l\right)}^{2}}{2\sigma_{d}^{2}}\right)$$ (3)

Eq 4 is the mathematical form of the range Gaussian function.


$$exp\left(-\frac{\left||I\left(i,j\right)-I{\left(k,l\right)}^{2}|\right|}{2\sigma_{r}^{2}}\right)$$ (4)

Spatial filtering convolves only the weight coefficient of the spatial distance (the closer to the center point, the larger the weight coefficient) with the image to determine the pixel value of the center point. Range filtering implies that the point whose pixel value is closer to that of the center point has a larger weight and that the point whose pixel value differs greatly possesses a smaller weight coefficient in the neighborhood. The range weight is determined by the Gaussian function range. Therefore, *σ*_*d*_ and *σ*_*r*_ directly affect the denoising ability of the filter. The larger the value of *σ*_*d*_ is, the greater the effect on each pixel, and the image will become more blurred. *σ*_*d*_ should be larger when there are fewer noise points. The larger *σ*_*r*_ is, the more obvious the blurring effect.

### Adaptive region growing algorithm

Image segmentation includes the technology and process of dividing an image into several nonoverlapping, meaningful, and identical feature areas to extract the target of interest. The features here could include the grayscale, color, texture, etc. Predefined targets could correspond to a single area or multiple areas [14].

The defects of ultrasonic phased array B-scan images are usually connected regions having similar features, such that the region growing method can be used to segment the images. The basic idea of traditional region growth is to find a seed point pixel for each region that needs to be split as the starting point for growth. Thus, pixels possessing the same or similar properties as the seed pixel in the neighborhood around the seed pixel (based on some predefined criteria for growth or similarity) are merged into the region where the seed pixel points are located. These pixels are used as new seed pixels to continue the above process until no more pixels that meet the conditions are included, after which a region is finally formed [15].

**Fig 2:**
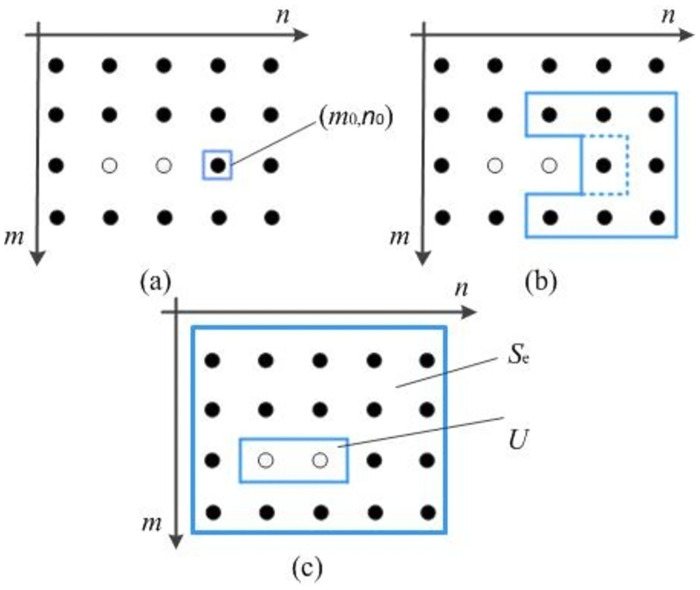
Schematic diagram of region growth in the B-scan image.

As shown in Fig 2, a schematic model of region growth in ultrasound images is established to better understand the basic theory of automatic region growth. The parameters and meanings of each variable are listed in Table 1 to easily distinguish between parameters.

**Table 1:** Definition of variables in the model.

No.	Variable names	Variable definition
**1**	*T(m*, *n)*	Matrix of B-scan images
**2**	*m*	The coordinates in the depth direction
**3**	*n*	The coordinates in the moving direction of the ultrasonic transducer
**4**	*T(m*_*0*_, *n*_*0*_*)*	The gray value at the point (*m*_*0*_, *n*_*0*_)
**5**	*U*	Nonseed region
**6**	*S*_*e*_	Seed region

As shown in Fig 2, there is an image matrix with 4 × 5 pixels and a grayscale of 1. Whether each pixel is black or white is used as a measure of its similarity. The steps of implementation of the region-growing method are as follows:

1. First, the starting seed point is set. The starting seed point is a point (*m*_0_, *n*_0_) in Fig 2(a);
2. The seed point characteristics are compared with the characteristics of the unclassified pixels in the neighborhood. If the characteristics are similar, they are merged into the same region to form a new seed point. In Fig 2(b), the pixel characteristics of point (*m*_0_, *n*_0_) and its 8 neighbor points are compared. If the colors are the same (both black), they will be merged into the same area as a new seed point;
3. Step (2) is repeated for new seed points until no new seed points can be merged into the region, and finally, Fig 2(c) is obtained. The ultrasonic B-scan image matrix *T*(*m*, *n*) is divided into two parts: non-seed region *U* and seed region *S*_*e*_.

Therefore, three problems need to be solved during the actual application of the region-growing method. The first is to determine a set of seed pixels (selecting seeds) that could correctly represent the desired region, the second is to determine the criteria for including adjacent pixels in the growth process (determination threshold), and the third is to confirm the conditions or rules for stopping the growth process (stopping conditions).

### Mathematical morphology processing method

Due to the geometric shape and coupling status of the complex components, problems such as divergence and false boundaries in the defects after segmentation persist, and thus, defect extraction and quantization accuracy are seriously affected. Therefore, an erosion algorithm is used for further optimization of the image after binarization [16].

The structuring element is selected as a 3 × 3 black point, and the erosion algorithm reduces the boundary of the object by one pixel along the perimeter. Edge detection is equivalent to corroding the original image with 9 points structuring elements of 3 × 3 blocks and then the original image minus the eroded image [17].

**T** is the image, and *B* is the structuring element. *B*_*z*_ represents the result after structural element *B* shifts to *Z*, *B*^*s*^ represents the symmetric set of structural elements to the point of origin, and the mathematical expression is as follows:

$$B^{s}=\left\{-b;b\in B\right\}$$ (5)

Then, the erosion operation is defined as:

$$\text{T}\Theta B^{s}=\left\{Z;B_{z}\subseteq \text{T}\right\}$$ (6)

The boundary operator extracted by mathematical morphology is:

$$ED\left(\text{T}\right)=\text{T}-(\text{T}\Theta E)$$ (7)

where *E* is 3 × 3 structural elements and *ED* (**T**) represents the boundary of image **T**.

The edge width of the object detected by this method is only one pixel; therefore, it has high positioning accuracy. The binarized image has complete object outlines; thus, the extracted edges are continuous.

## Algorithm implementation

**Fig 3:**
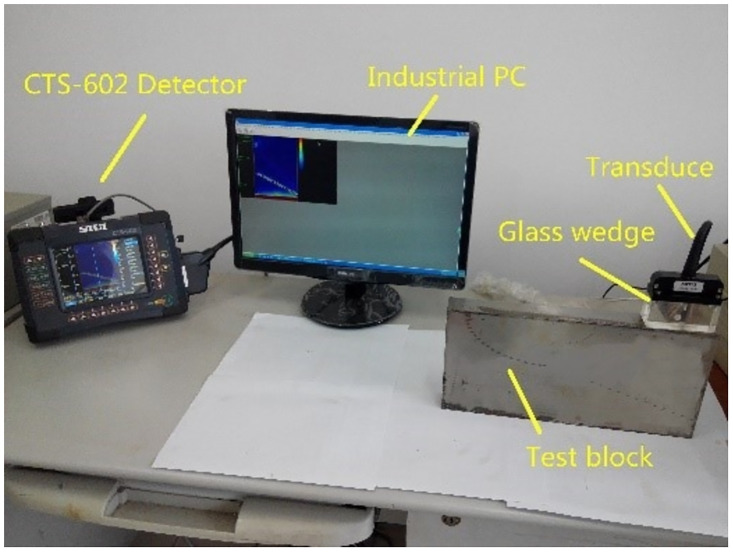
Actual diagram of the B-type test block detection.

**Fig 4:**
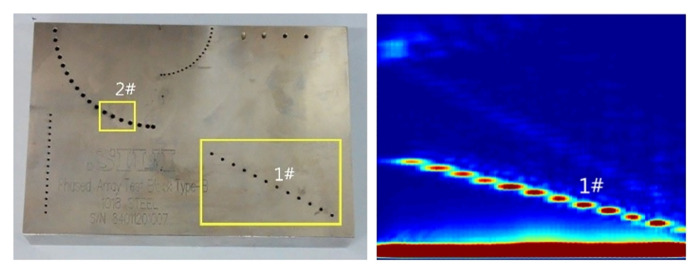
Physical drawing of the type B test block and original detection image.

The implementation of the improved adaptive region growing algorithm plays a pivotal role in effectively segmenting connected regions sharing characteristics with the seed growth point, thereby providing precise regional boundary information. Fig 3 presents the physical setup of the experimental device. Utilizing the CTS-602 device, ultrasonic phased array B-scan imaging is conducted on the defect area of an oblique through-hole in the phased array B-shaped test block. The detection depth at hole #1 is set at 20 mm, where the optimal echo amplitude is achieved when the focusing area of the ultrasonic phased array coincides with the detection site. Consequently, a focusing depth of 20 mm is selected to maximize detection capability. The choice of transducer hinges on its focusing proficiency. The TL5F64 transducer with a dynamically adjustable near-field depth ranging from 28.63 mm to 83.3 mm is chosen. Prior research indicates that the best detection results are obtained near the near-far field boundary [18]. Hence, a near-field depth of 28.63 mm is selected, corresponding to a transducer aperture of 20 mm. To balance aspects like acoustic attenuation, focusing performance, and transducer resolution, a center frequency of 5 MHz is chosen for the transducer. Fig 4 illustrates the Phased array B-type test block along with the original detection image.

**Fig 5:**
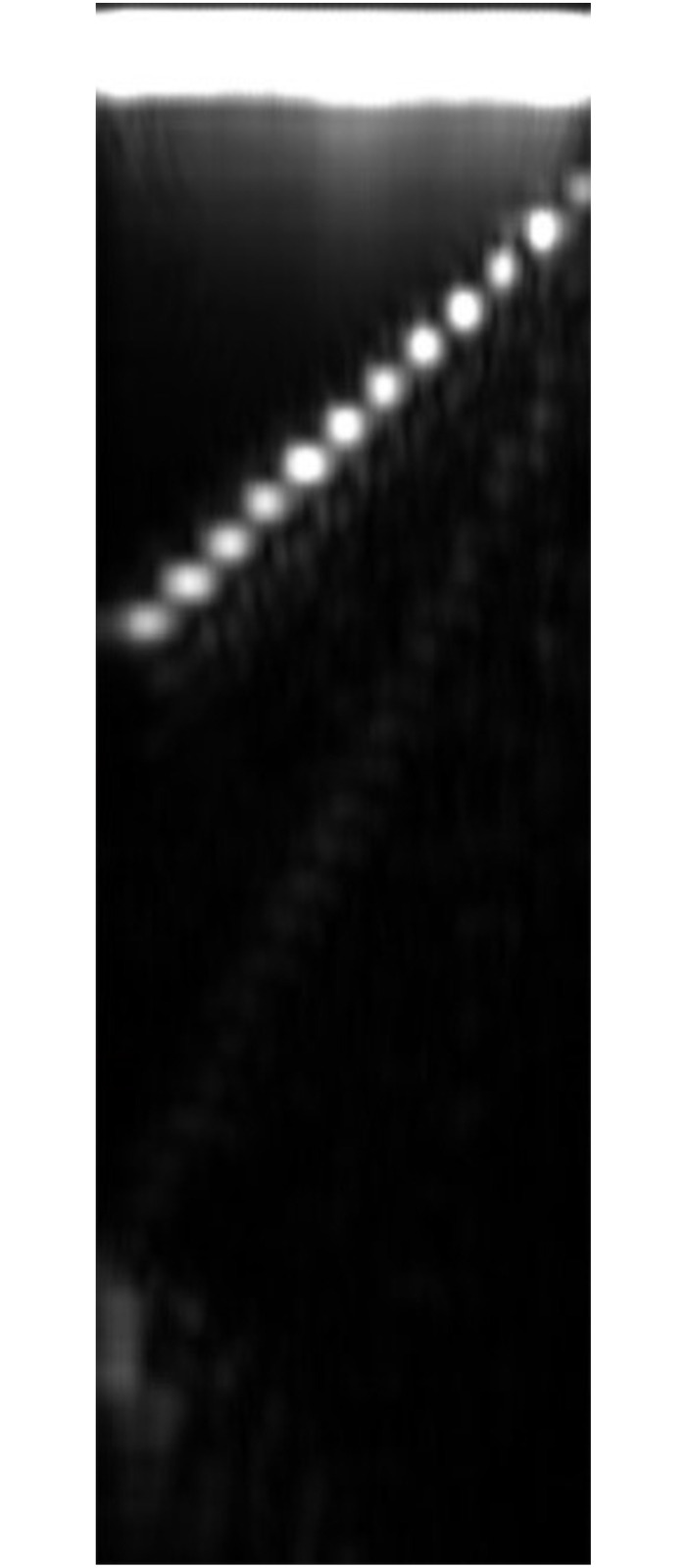
Grayscale image.

The original detection image is comprised of the background, target, and noise clutter. The presence of redundant information, such as noise and clutter, impedes defect detection. Additionally, artifacts in the background can blur defect boundaries, significantly affecting defect feature extraction. The initial step involves processing the original image using the weighted average method, where each component generates weights automatically and performs weighting operations based on the significance of each component on the pixel and other indicators. The resulting grayscaled image is shown in Fig 5.

**Fig 6:**
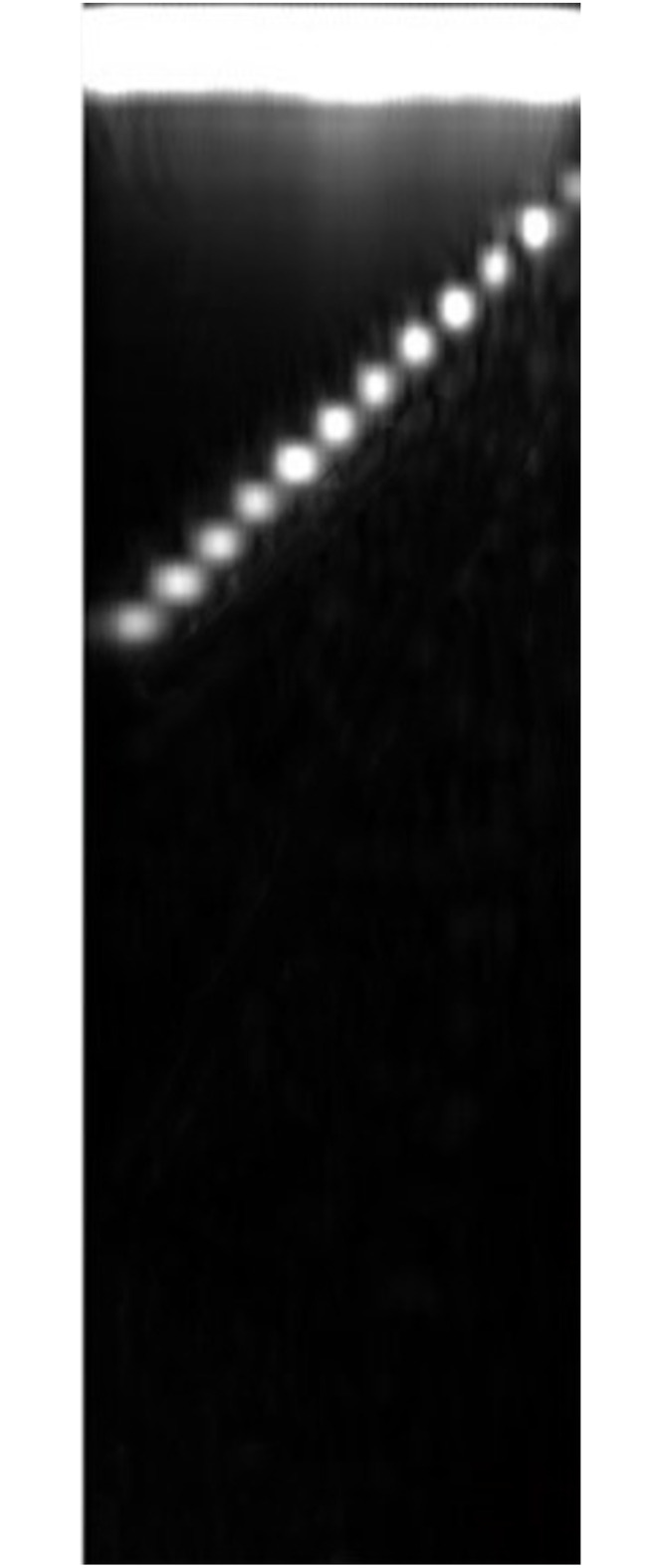
Detected image after bilateral filtering.

Despite processing, serious noise interference persists in the grayscaled image. Bilateral filtering is employed to enhance the signal-to-noise ratio and filter out noise. In this process, a larger standard deviation in calculations leads to a smaller weight difference, implying less emphasis on this factor. Conversely, *σ*_*d*_ indicating spatial domain smoothness, is crucial for areas lacking sharp edges or exhibiting gradual transitions. An increase in *σ*_*d*_ helps eliminate noise in these regions. However, if *σ*_*d*_ exceeds 1, the image’s detail processing capability starts to diminish, thus *σ*_*d*_ is set at 1.*σ*_*r*_ relates to the range of value differences, where reducing *σ*_*r*_ accentuates edge definition, resulting in a clearer image. Utilizing Laplace for fast standard deviation estimation, optimal results are achieved with *σ*_*r*_ set at 0.1 [19]. The local intensity sliding window is typically not more than 11 × 11 pixels and is always an odd number. In this study, it is uniformly set to 11 × 11 to boost smoothness and computational efficiency. Care is taken in selecting the filter half-width N, as a larger value can over-smooth edges and target regions, hindering gradient vector detection. Consequently, N is finalized at 5, based on the sliding window size. Fig 6 displays the grayscale image post bilateral filtering.

The detailed information of the image can not only be preserved but can also achieve a good filtering effect after adopting bilateral filtering. Due to the defect image in the processed ultrasonic phased array B-scan image, it does not connect with other areas. The region growing method is used for feature image segmentation to effectively segment the connected regions with the same features as defect points and provide good region boundary information.

The most critical aspect of the adaptive region growing algorithm is to determine the growth starting point of the growth criteria. The reflected signals of defects generally appear as prominent color values in ultrasonic phased array B-scan images. For example, aiming at a 256-level grayscale image, the higher the amplitude of the defect signal is, the brighter the color. Therefore, while determining the defect region, the prominent color on the B-scan image must first be traversed, which is equivalent to the seed pixel in the region growth method, and then the defect signal region from the inside and outside should be determined.

**Fig 7:**
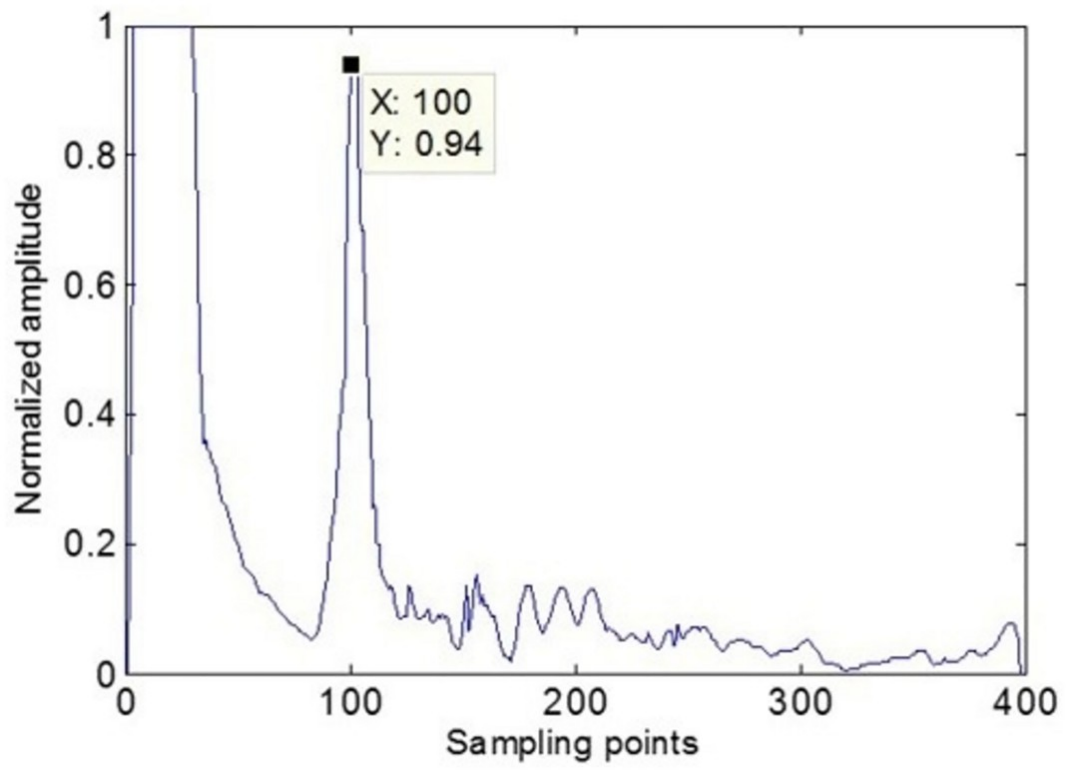
The a-wave amplitude at the scan line.

**Fig 8:**
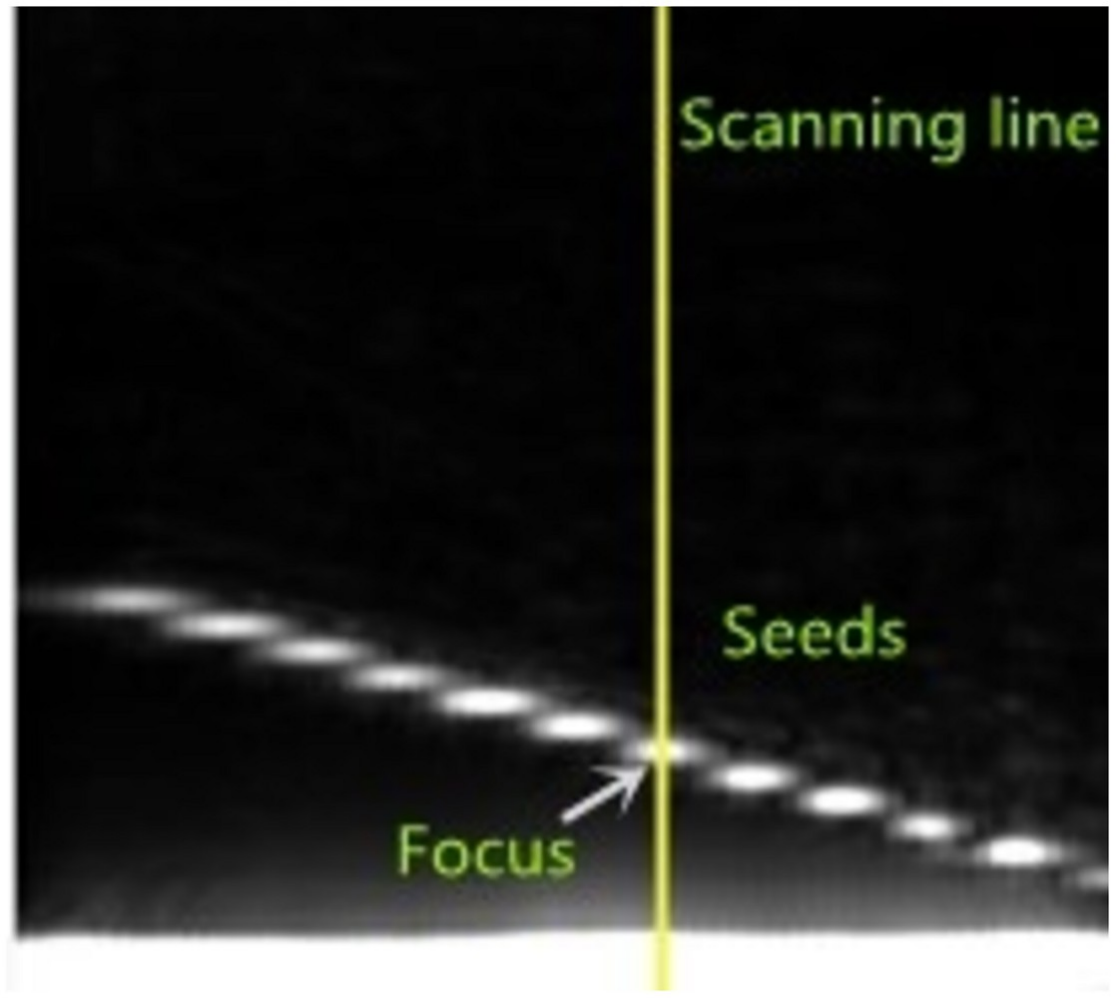
Schematic diagram of initial seed point selection.

The position of the scan line was adjusted such that it lies in the center of the defect, and the A-wave detection data at the scan line in the ultrasonic phased array B-scan image were extracted. As shown in Fig 7, the defect signal is reflected in the form of peaks. Usually, the pixel with a prominent color value in the ultrasonic B-scan image is selected as the seed pixel, which is at the maximum amplitude. Since the amplitude of the initial wave is higher than the defect echo, the focal length can be set at the defect depth to automatically and accurately locate the seed point. As shown in Fig 8, the echo amplitude at the defect is the largest during this time, and there will be no interference with the initial wave.

**Fig 9:**
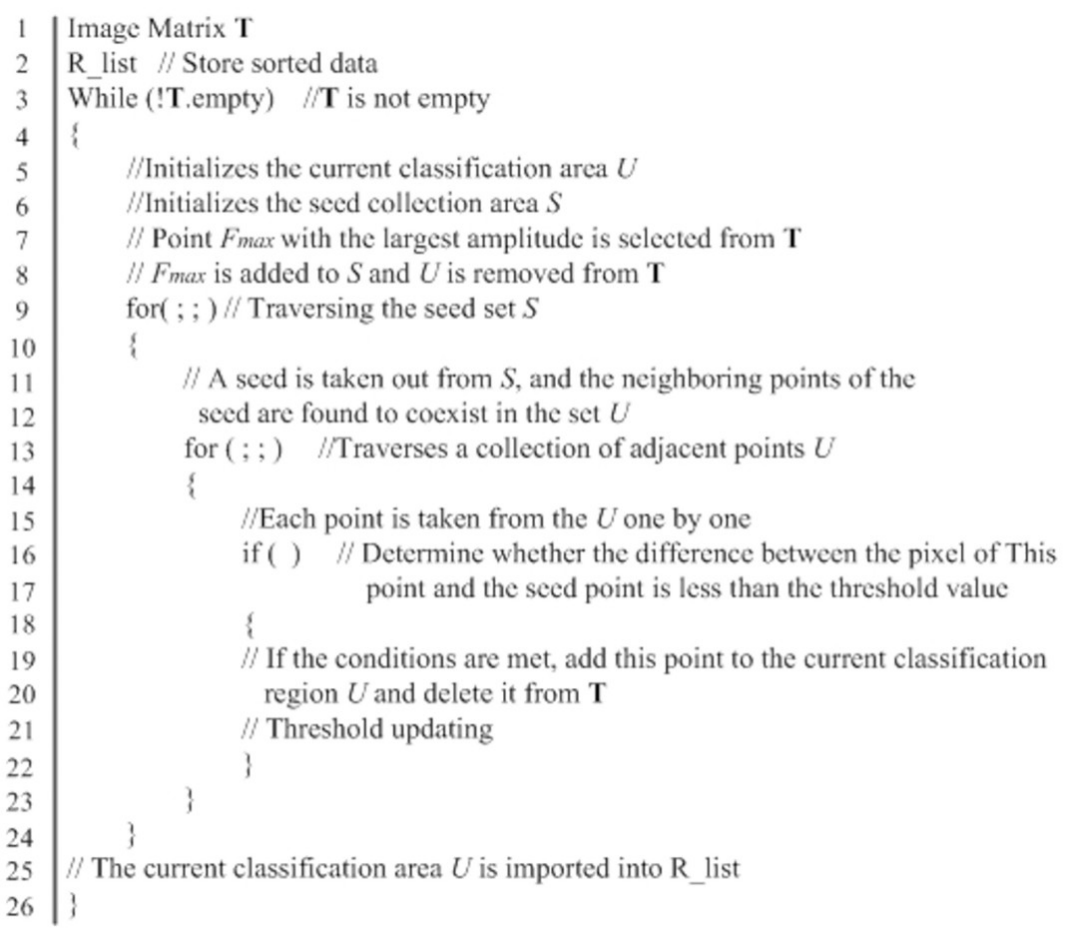
Pseudocode of the adaptive region growing algorithm in this paper.

The seed point is compared with its 8 neighborhood pixels (vertical, horizontal, and 2 diagonal) by using a 3 × 3 sliding window *G*. The average pixel value of the window corresponding to each point is calculated in the region of interest as follows:

$$M\left(f\left(i,j\right)\right)=\frac{\sum_{\left(x,y\in G\right)}f\left(x,y\right)}{N\times N}\;f\left(i,j\right)\in I$$ (8)

where *f(i*,*j)* is the pixel point in the region of interest; *f(x*,*y)* is the pixel point in the window; *N* is the sliding window size; *I* is the image window area, and the threshold is set as *T*_*m*_. Thus:

$$T_{m}=c\cdot \frac{1}{K}\sum_{f\left(i,j\right)\epsilon I}M\left(f\left(i,j\right)\right)$$ (9)

where *C* is a constant coefficient, and *C* is set to 2 by consulting references [20] in this paper; *K* is taken as 9, which is the total number of pixels in the region of interest *I*. The growth conditions were determined from the regional growth of the initial seed point. The gray value is one of the main features used to distinguish the target from the background in ultrasound phased array B-scan images. The absolute value of the gray value difference (*D* = |*f*_*1*_—*f*_*2*_|) between two adjacent pixels is used as the similarity criterion. The threshold *T* is set, and the pixels in the neighborhood around the seed point are searched. If *D*≤*T*, the point must be grown, and it should be used as a seed point to continue to grow. Among them, the threshold *T*_*m*_ is kept dynamically updated during the sliding process of window *G*. Next, the average gray value of the area where the new pixel is located is compared with the gray value of the pixels in each area, and growth is stopped until it reaches the boundary or the points that no longer meet the conditions. The error caused by the transition and merging growth of different regions in the region where the gray level changes slowly is overcome by adopting this method. Fig 9 shows the algorithm pseudocode.

A suitable initial threshold must be set before the growth of ultrasound phased array B-scan images, as it will result in execution failure of the algorithm if the threshold is chosen to be too large or too small. Studies have shown that selecting the average gray value of the B-scan image after preprocessing as the initial threshold *T* could obtain a better segmentation effect. The average grayscale value of the target image can be calculated from the original data to be 35. Therefore, *T* is selected as 35. Table 2 compiles all the parameters involved in the image processing procedure.

**Table 2:** Key parameters in image processing.

No.	Variable names	Value	Variable definition
1	σ_*d*_	The smoothness of the spatial domain	1
2	*σ*_*r*_	The difference in the value range	0.1
3	*N*	The half-width of the filter	5
4	*n*	The local intensity sliding window	11×11
5	*c*	Constant coefficient	2
6	*K*	The total number of pixels in the region of interest I	9
7	*T*	The initial threshold	35

The specific algorithm process is as follows.

**Fig 10:**
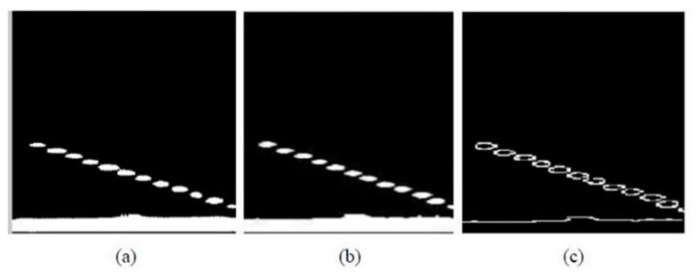
Mathematical morphological processing. (a) The binarized image after segmentation; (b) The image after the closed operation; (c) The image marked by the Sobel operator.

1. A 3 × 3 sliding window is used to traverse the ultrasound B-scan image. The average value of the window grayscale is calculated, and the point with the largest amplitude of the focal length position is selected as the initial seed point.
2. The unmarked pixels in the surrounding 8 neighborhoods are searched sequentially, starting from the seed point. It needs to be judged whether it is a boundary point and if it satisfies the growth condition *D*≤*T*. If it meets the growth conditions and is not a boundary point, the pixel is marked.
3. The seed points are continuously updated, the marked points are regarded as new seed points, the average gray value of the window is recalculated as the updated window threshold *T*_*m*_, new pixel points are obtained, and the average gray value of the area where the new pixel is located is compared with each grayscale value of the domain pixels. If the seed points have been increased, return to step 2; if not, proceed with the next step.
4. The marked points are separated from the image and segmented, and the binarized image after segmentation is shown in Fig 10(a).

It is necessary to erode and dilate the image post-binarization to extract defect features more accurately. Erosion and dilation are two basic morphological operations that implement the resulting elements in an input image and generate a corresponding output image. The dilation operation convolves the input image with some kernels. The maximum pixel value overlapped by the kernels is calculated and used for replacing the image pixels at the anchor points during the scanning of kernels on the image. The entire operation will expand the highlighted area in the image, and the image dilation operation process follows the following formula:

$$dst\left(x,y\right)={\text{max}}_{\left(x^{\prime }+y^{\prime }\right):element\left(x^{\prime }+y^{\prime }\right)\neq 0}src\left(x+x^{\prime },y+y^{\prime }\right)$$ (10)

The process of the erosion operation calculates local minima in the kernel region. The minimum pixel value of kernel overlap is calculated while scanning the kernel, and this value is used to replace the image pixel of the anchor point.


$$dst\left(x,y\right)={\text{min}}_{\left(x^{\prime }+y^{\prime }\right):element\left(x^{\prime }+y^{\prime }\right)\neq 0}src\left(x+x^{\prime },y+y^{\prime }\right)$$ (11)

The closed operation is used for filling the small holes in the target, connecting the disconnected adjacent targets, and smoothing the edges without significantly changing the area. Fig 10(b) shows an image processed by the closed operation. The Sobel operator is used for edge extraction to facilitate the subsequent extraction of area, perimeter, and other features; it can produce a better detection effect, smooth the noise, and provide more accurate edge direction information [21]. Fig 10(c) shows the corresponding results.

## Experimental analysis

**Fig 11:**
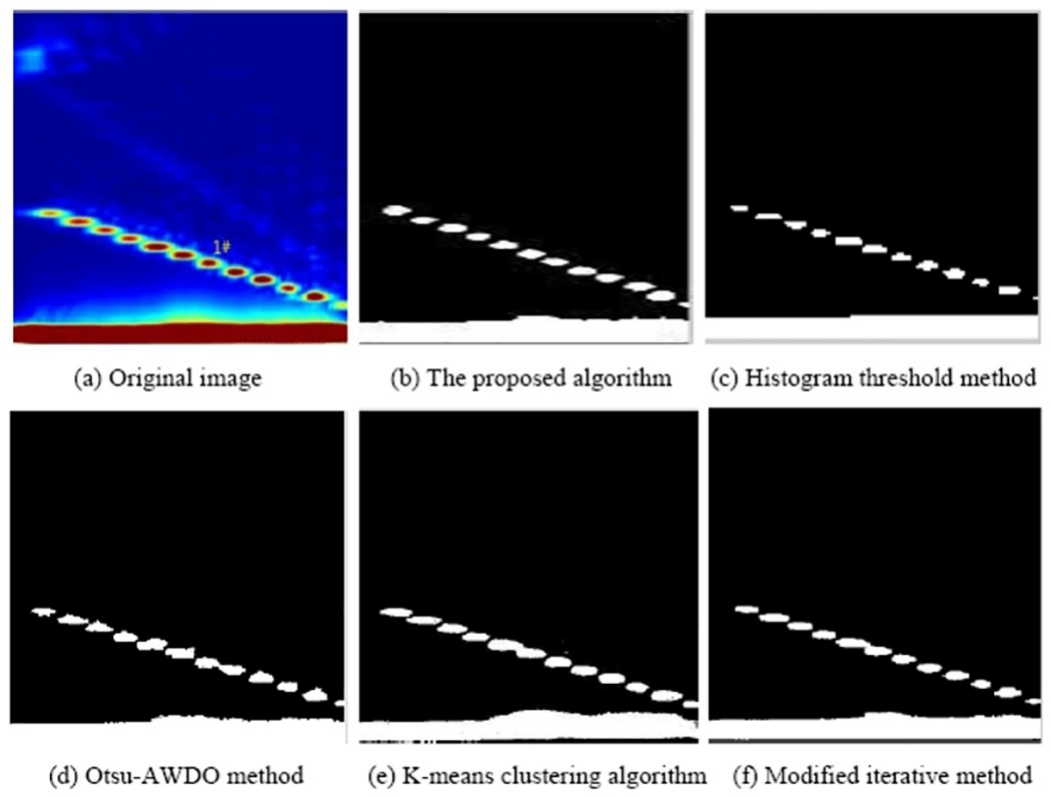
1# detection images processed by different methods. (a) Original image; (b) The proposed algorithm; (c) Histogram threshold method; (d) Otsu-AWDO method; (e) K-means clustering algorithm; (f) Modified iterative method.

**Fig 12:**
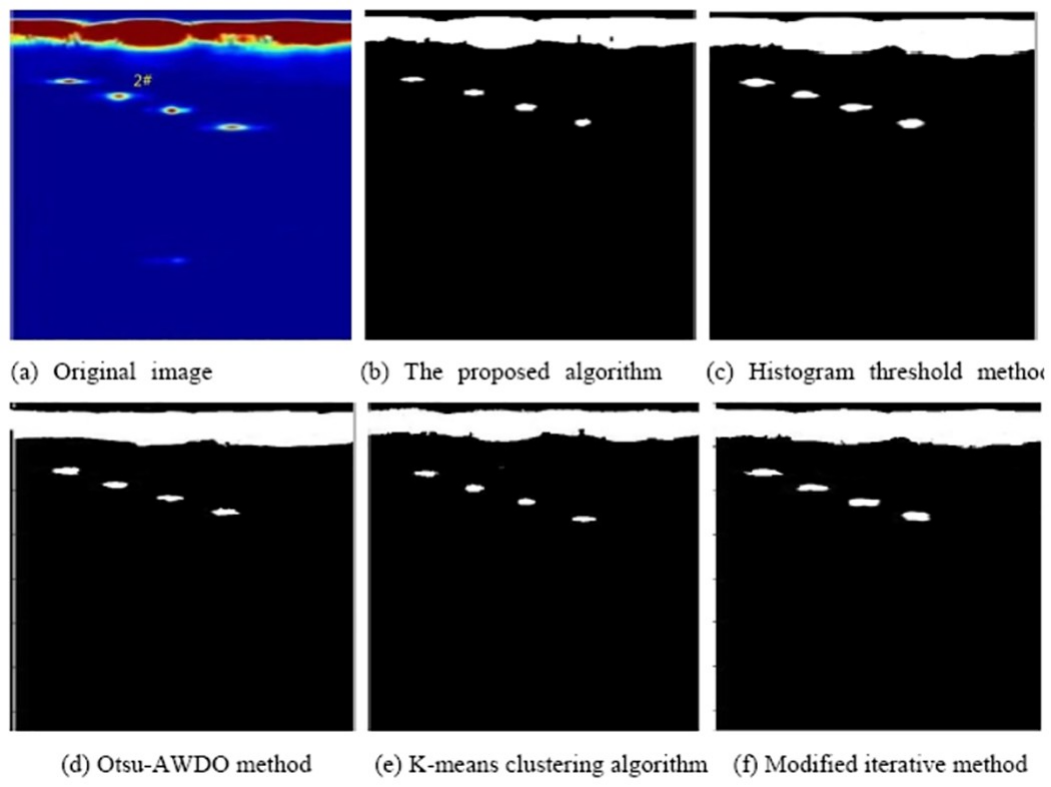
2# detection images processed by different methods. (a) Original image; (b) proposed algorithm; (c) histogram threshold method; (d) Otsu-AWDO method; (e) K-means clustering algorithm; (f) modified iterative method.

The proposed method will be compared with the current effective defect feature extraction algorithm of ultrasonic phased array B-scan images to verify the effectiveness of the proposed method. The histogram threshold method [6], the Otsu-AWDO method [5], the K-means clustering algorithm [7], and the modified iterative method [9] are used to process the detection image of hole #1 and defect #2 to obtain the optimal defect extraction method of the ultrasonic phased array B-scan image more accurately. The results are shown in Figs 11 and 12, respectively.

The noise and clutter in all final images have been basically filtered out according to the analysis of the #1 and #2 detection images processed by different methods. The final image is clear, and the shape of the target image is complete, which is beneficial to the subsequent feature extraction. However, it can be seen from Fig 11 that the target image processed by the histogram threshold method has slender gullies and incomplete contour lines, while the target images processed by the Otsu-AWDO and K-means algorithms have narrow discontinuities. The target defect contours in Fig 12(c) and 12(d) show some bending and pitting, whereas the target images in Fig 12(e) and 12(F) show greater deroundness. The proposed algorithm can extract defect features #1 and #2 with high revivification and continuous boundaries.

The pixel number of each defect feature can be obtained from Figs 11 and 12. The pixels of the feature value are further compared with the total pixels of the whole area. The actual value of the feature is obtained by multiplying the image scaling. The defect area and perimeter are extracted to evaluate the quantitative ability of defects, and the perimeter-to-area ratio, major axis, and minor axis values are extracted to evaluate the qualitative situation of defects. The detailed defect feature values of defect holes #1 and #2 extracted by different methods are shown in Table 3. Thus, the relative error *δ* can be obtained according to formula 12.

$$\delta =\frac{\Delta }{L}\times 100\%$$ (12)

where *Δ* is the absolute error and *L* is the real value.

**Table 3:** Defect characteristic values extracted by different methods.

Defect S/N	Defect characteristics	Defect area/mm^2^	Defect perimeter/mm	Ratio of perimeter to area	Major axis/mm	Minor axis/mm
**1#**	Actual value	1.77	4.71	2.66	1.5	1.5
	The proposed algorithm	**1.98(11.8%)**	**5.19(10.2%)**	**2.62(1.9%)**	1.60(6.7%)	1.63(8.6%)
	Histogram threshold method	1.21(32.4%)	3.95(16.1%)	3.26(22.1%)	1.33(11.3%)	1.37(8.7%)
	Otsu-AWDO method	2.12(12.8%)	6.21(31.8%)	2.93(9.7%)	1.75(16.7%)	1.72(14.6%)
	K-means clustering algorithm	2.35(31.3%)	5.87(23.4%)	2.49(6.4%)	1.68(12%)	1.64(9.3%)
	Modified iterative method	2.16(20.6%)	5.51(16.9%)	2.55(4.5%)	**1.59(6%)**	**1.56(4%)**
**2#**	Actual value	3.14	6.28	2	2	2
	The proposed algorithm	**3.52(12.1%)**	6.87(9.4%)	**1.88(6%)**	**2.12(6%)**	**2.17(8.5%)**
	Histogram threshold method	4.31(33.1%)	7.42(18.2%)	1.72(14%)	2.75(38%)	2.61(30.5%)
	Otsu-AWDO method	3.63(15.6%)	8.09(28.8%)	2.23(11.5%)	2.33(16.5%)	2.20 (10%)
	K-means clustering algorithm	3.71(14.5%)	**6.59(4.9%)**	1.78(11.2%)	2.24(12%)	2.31(15.5%)
	Modified iterative method	4.45(37.3%)	7.68(22.3%)	1.73(13.5%)	3.05(53%)	2.91(45.5%)

Table 3 reveals the dimensions of defects #1 and #2 as determined by the algorithm. The major axes measure 1.60 mm and 2.12 mm, while the minor axes are 1.63 mm and 2.17 mm, respectively. These values exhibit a slight overestimation compared to the actual defect size of 1.5 mm. The algorithm calculates the areas of these defects as 1.98 mm^2^ and 3.65 mm^2^, respectively, indicating minor deviations from the true sizes. Figs 11 and 12 demonstrate that the algorithm successfully retains the detailed contours of the defects, ensuring well-defined boundaries and accurately representing their fundamental shapes and sizes during the eigenvalue extraction process.

The improved Otsu method demonstrates enhanced accuracy in extracting defect areas, with area errors of 12.8% and 15.6% for defects #1 and #2, respectively. However, this method yields unclear defect boundaries, leading to significant errors in contour circumference. Iterative methods, used for extracting basic features of small defects, provide relatively complete boundary information. The extracted perimeter-to-area ratio for defect #1 is 2.55 (compared to the actual value of 2.67), but the relative error in feature extraction for larger defects (approximately 2 mm in diameter) is notably higher, reaching 1.73 (against the actual value of 2). The k-means clustering algorithm shows better performance for larger defects, while the histogram threshold method is less accurate for defect detection. The feature error for each defect is considerable using these methods, and the contour often undergoes significant deformation, which is not conducive to quantitative and qualitative defect analysis.

A comprehensive analysis indicates that the relative errors in defect areas and perimeter-to-area ratios extracted by the adaptive region growth method are below 13% and 6%, respectively, with the relative error of major and minor axes under 9%. The relative errors of all defect quantitative eigenvalues extracted by this algorithm are lower than those obtained using the histogram threshold method, Otsu-AWDO method, K-means clustering algorithm, and modified iterative method. The detection image processed by the proposed algorithm and the extracted defect feature values accurately reflect the actual size and shape of defects, allowing for more precise defect extraction and quantification.

## Study limitations

The use of bilateral filtering for noise reduction in the initial phase is particularly effective in middle and low-frequency domains, preserving crucial contour details in the target image. However, in high-attenuation materials or heterogeneous media, where ultrasonic phased array techniques require increased transmission frequency for improved sound wave penetration, bilateral filtering may struggle to fully eliminate high-frequency noise. This could result in undesired artifacts in the resultant image. To address this issue, more advanced techniques, such as New Reverse Time Migration [22], must be employed in conjunction with bilateral filtering. Additionally, it is worth noting that the lattice Boltzmann method offers a valuable approach for optimizing transducer parameters and enhancing the overall quality of ultrasonic phased array imaging [23].

## Conclusion

Ultrasonic phased array B-scan images differ significantly from traditional ultrasonic detection images, particularly in terms of gray level differentiation and the overlapping of the initial wave, defect echo, bottom echo, and noise. Traditional image segmentation methods like the histogram threshold, Otsu method, K-means clustering algorithm, and modified iteration method face challenges in these contexts due to poor filtering effects, fuzzy detail processing, and unclear boundary recognition, leading to inaccuracies in defect feature extraction.

In this study, the noise-mixed ultrasonic phased array B-scan image is first converted to grayscale, followed by bilateral filtering to retain image details while filtering noise. The scanning line position is adjusted in real-time, and the A-wave data of the defect scanning line are extracted to set regional growth seed points automatically. The 3 × 3 moving window technique is employed for traversing all areas, continuously updating seed points and window thresholds for accurate defect image segmentation. Finally, the combination of closed operation and Sobel operator, based on mathematical morphology, is used for defect edge and feature extraction. The results demonstrate the algorithm’s superiority in segmenting and extracting ultrasonic phased array B-scan images of defects such as holes.

The segmented and extracted ultrasonic B-scan defect images exhibit distinct edges and accurate features, laying a solid foundation for subsequent identification and analysis of acoustic defects. Future work will focus on improving device instrumentation for more precise evaluation of tumors and lesions in medical ultrasound, aiding in diagnosis and prognosis of circulatory system tumors and lesions, and enabling detection and analysis of surface and internal defects in composite materials and special equipment using machine vision technology.

## Supporting information

S1 File (ZIP)
